# Effect of *Haemophilus influenzae* type b vaccination without a booster dose on invasive *H influenzae* type b disease, nasopharyngeal carriage, and population immunity in Kilifi, Kenya: a 15-year regional surveillance study

**DOI:** 10.1016/S2214-109X(15)00316-2

**Published:** 2016-02-05

**Authors:** Laura L Hammitt, Rosie J Crane, Angela Karani, Alex Mutuku, Susan C Morpeth, Polly Burbidge, David Goldblatt, Tatu Kamau, Shahnaaz Sharif, Neema Mturi, J Anthony G Scott

**Affiliations:** a Department of Epidemiology and Demography, KEMRI-Wellcome Trust Research Programme, Centre for Geographic Medicine Research–Coast, Kilifi, Kenya; b Department of International Health, Johns Hopkins Bloomberg School of Public Health, Baltimore, MD, USA; c Nuffield Department of Clinical Medicine, University of Oxford, Oxford, UK; d Department of Infectious Disease Epidemiology, London School of Hygiene & Tropical Medicine, London, UK; e Institute of Child Health, University College London, London, UK; f Kenya Ministry of Public Health and Sanitation, Kilifi, Kenya

## Abstract

**Background:**

*Haemophilus influenzae* type b (Hib) conjugate vaccine, delivered as a three-dose series without a booster, was introduced into the childhood vaccination programme in Kenya in 2001. The duration of protection and need for a booster dose are unknown. We aimed to assess vaccine effectiveness, the impact of the vaccine on nasopharyngeal carriage, and population immunity after introduction of conjugate Hib vaccine in infancy without a booster dose in Kenya.

**Methods:**

This study took place in the Kilifi Health and Demographic Surveillance System (KHDSS), an area of Kenya that has been monitored for vital events and migration every 4 months since 2000. We analysed sterile site cultures for *H influenzae* type b from children (aged ≤12 years) admitted to the Kilifi County Hospital (KCH) from Jan 1, 2000, through to Dec 31, 2014. We determined the prevalence of nasopharyngeal carriage by undertaking cross-sectional surveys in random samples of KHDSS residents (of all ages) once every year from 2009 to 2012, and measured Hib antibody concentrations in five cross-sectional samples of children (aged ≤12 years) within the KHDSS (in 1998, 2000, 2004–05, 2007, and 2009). We calculated incidence rate ratios between the prevaccine era (2000–01) and the routine-use era (2004–14) and defined vaccine effectiveness as 1 minus the incidence rate ratio, expressed as a percentage.

**Findings:**

40 482 children younger than 13 years resident in KHDSS were admitted to KCH between 2000 and 2014, 38 206 (94%) of whom had their blood cultured. The incidence of invasive *H influenzae* type b disease in children younger than 5 years declined from 62·6 (95% CI 46·0–83·3) per 100 000 in 2000–01 to 4·5 (2·5–7·5) per 100 000 in 2004–14, giving a vaccine effectiveness of 93% (95% CI 87–96). In the final 5 years of observation (2010–14), only one case of invasive *H influenzae* type b disease was detected in a child younger than 5 years. Nasopharyngeal *H influenzae* type b carriage was detected in one (0·2%) of 623 children younger than 5 years between 2009 and 2012. In the 2009 serosurvey, 92 (79%; 95% CI 70–86) of 117 children aged 4–35 months had long-term protective antibody concentrations.

**Interpretation:**

In this region of Kenya, use of a three-dose primary series of Hib vaccine without a booster dose has resulted in a significant and sustained reduction in invasive *H influenzae* type b disease. The prevalence of nasopharyngeal carriage is low and the profile of Hib antibodies suggests that protection wanes only after the age at greatest risk of disease. Although continued surveillance is important to determine whether effective control persists, these findings suggest that a booster dose is not currently required in Kenya.

**Funding:**

Gavi, the Vaccine Alliance, Wellcome Trust, European Society for Paediatric Infectious Diseases, and National Institute for Health Research.

## Introduction

Inclusion of *Haemophilus influenzae* type b (Hib) conjugate vaccine in the routine infant immunisation programme has led to tremendous reductions in childhood *H influenzae* type b morbidity and mortality in both developed and developing countries.^1^, ^2^ Hib vaccine was introduced into the Kenyan childhood Expanded Program on Immunization (EPI) in November, 2001, as a three-dose series administered at 6, 10, and 14 weeks of age. Within 3 years of introduction, invasive *H influenzae* type b disease had decreased to 12% of its baseline level.^3^ A booster dose of Hib vaccine is not included in the Kenyan EPI schedule, nor in the schedules of 72 (92%) of 78 low-income and lower-middle-income countries.^4^

In the UK, 10 years after the introduction of the Hib primary vaccination, waning levels of antibody to polyribosylribitol phosphate (PRP)—an *H influenzae* type b polysaccharide capsule component—as well as persistence of *H influenzae* type b nasopharyngeal colonisation and rising rates of invasive disease, prompted introduction of a booster dose of Hib vaccine for children aged 12–15 months in 2006.^5^, ^6^ The Government of Mexico also introduced a booster dose of Hib vaccine 9 years after launching the primary vaccination programme, in part because of waning anti-PRP antibodies in children aged 12–59 months.^7^ However, persistently low incidence of *H influenzae* type b meningitis in the western region of The Gambia more than a decade after Hib vaccine introduction shows that the disease can be adequately controlled in the absence of a booster dose.^8^

**Research in context**

**Evidence before this study**

We searched PubMed with the terms “Hib”, “*Haemophilus influenzae* type b”, “vaccine”, “effectiveness”, “seroepidemiology”, “anti-PRP”, “booster”, “cross reactive”, “carriage”, and “colonization” for articles published in any language before May 31, 2015. To identify additional publications we searched the reference lists of retrieved articles. More than a decade after conjugate *Haemophilus influenzae* type b (Hib) vaccines became available, only 2% of the global *H influenzae* type b disease burden was being prevented by vaccination. In 2001, Gavi, the Vaccine Alliance, offered financial support for the introduction of Hib vaccine in developing countries, and Kenya became one of the first African countries to include Hib vaccine in the national immunisation schedule. Like the vast majority of low-income and lower-middle-income countries, Kenya used a three-dose primary series of Hib vaccine, without a booster dose. A three-dose schedule without a booster is highly effective in reducing the burden of *H influenzae* type b disease in the short term; however, whether a booster dose is required to achieve sustained disease control is unclear. Although data from some countries have prompted the addition of a booster dose, other data show good control of *H influenzae* type b disease in the absence of a booster. The need for a booster dose of Hib vaccine is probably affected by local epidemiology and factors such as the potential for natural boosting.

**Added value of this study**

This study provides new data documenting the near elimination of invasive *H influenzae* type b disease in Kilifi, Kenya, in the 12 years after introduction of vaccine into the routine infant vaccination schedule without a booster dose. The detailed seroepidemiology work before and after vaccine introduction shows that the vaccine has led to improvements in population immunity in the youngest, highest-risk age groups without compromising immunity in older children.

**Implications of all the available evidence**

This study delivers compelling evidence of the long-term operational impact of a three-dose primary series of Hib vaccine in a low-income country and provides a clear answer to a pertinent policy question in Kenya: a booster dose of vaccine is not currently needed to control *H influenzae* type b disease.

The long-term effectiveness of a primary series of Hib vaccine in infancy can be inferred from incidence of invasive *H influenzae* type b disease, nasopharyngeal carriage prevalence, and seroepidemiological data from the general population. Hib vaccination induces serum antibody production and reduces the nasopharyngeal carriage prevalence of *H influenzae* type b, thereby diminishing the risk of invasive disease. Reductions in carriage also reduce transmission of Hib between individuals. This contributes to herd protection, but also limits the opportunity for intermittent natural boosting of serological immunity. The pattern of *H influenzae* type b serological immunity in different age groups across time and the persistence of *H influenzae* type b serological immunity throughout the years of highest risk for *H influenzae* type b disease are likely to be important determinants of vaccine effectiveness beyond the primary vaccination period.

There is equipoise in the scientific community regarding the need for a booster dose of Hib vaccine to control disease in the long term.^9^ Herein we report vaccine effectiveness, the impact of the vaccine on nasopharyngeal carriage of *H influenzae* type b, and population immunity to *H influenzae* type b in the 13 years after introduction of conjugate Hib vaccine in infancy without a booster dose in Kenya.

## Methods

### Population

This surveillance study took place in the Kilifi Health and Demographic Surveillance System (KHDSS), a rural community on the Kenyan coast covering an area of 891 km^2^.^10^ A census of the KHDSS in 2000 defined the resident population and, since 2000, fieldworkers have been monitoring migration events by visiting every participating household roughly every 4 months. The annual population was 199 732 in 2000, 239 396 in 2007, and 279 877 in 2014. The population is served by several government-funded health centres and by one government hospital, Kilifi County Hospital (KCH). Among women attending antenatal care at KCH, the prevalence of HIV infection ranged between 2·4% and 4·6% during 2005–13, with a general downwards trend. The prevalence of HIV in children in Kenya was estimated in 2012 to be 0·9% nationally.^11^

On Nov 1, 2001, the Government of Kenya introduced tetanus-toxoid-conjugated Hib vaccine as part of a pentavalent formulation in which lyophilised Hib vaccine (Hiberix; GlaxoSmithKline, Rixensart, Belgium) was resuspended in the diphtheria, tetanus, whole-cell pertussis, hepatitis B vaccine (Tritanrix, GlaxoSmithKline). The first children eligible to receive a 6-week dose of this pentavalent vaccine were born on Sept 20, 2001, and would have been eligible to receive their third dose at the end of December, 2001.

The protocol was approved by the Oxford Tropical Ethical Review Committee (No. 30-10) and the Kenya National Ethical Review Committee (SSC1433). Parents or guardians of all study participants provided written informed consent.

### Assessment of vaccine effectiveness

To assess vaccine effectiveness, we determined the prevalence of invasive *H influenzae* type b disease in children aged 12 years or younger admitted to KCH between Jan 1, 2000, and Dec 31, 2014. Blood samples are routinely taken for culture at the time of admission (except for trauma patients or patients admitted for elective surgery). Blood was cultured using an automated BACTEC instrument (BD Diagnostics, Franklin Lakes, NJ, USA). From 1998 to 2014, with the exception of a brief change in practice in 2004–05, the clinical indications for lumbar puncture were impaired consciousness or meningism in children younger than 5 years, prostration in children younger than 3 years, seizures (other than febrile seizures) in children younger than 2 years, and suspicion of sepsis in children younger than 60 days. Cerebrospinal fluid (CSF) was cultured on horse blood and chocolate agar. Beginning in 2003, HIV testing was done on the blood of children admitted to KCH according to the Kenya national policy for paediatric hospital admissions, using two rapid antibody tests. Treatment for all disorders was according to WHO guidelines at the time of admission.

Isolates of *H influenzae* from sterile-site cultures were identified by colony morphology, Gram stain, and X and V factor dependence at the KEMRI-Wellcome Trust laboratory, located adjacent to KCH. Capsular type was identified by PCR using either the *cap* locus (done by the Haemophilus Reference Unit/WHO Collaborating Centre for *Haemophilus influenzae,* Respiratory and Systemic Infection Laboratory, Health Protection Agency Centre for Infections, London, UK, for isolates collected in 2000–04) or the *bex*A locus (done by the KEMRI-Wellcome Trust laboratory in Kilifi, Kenya, for isolates collected in 2005–13).^12^ We defined a case of invasive *H influenzae* type b disease as isolation of type b *H influenzae* from a sterile-site culture in a child aged 12 years or younger admitted to KCH.

### Assessment of nasopharyngeal carriage

We investigated nasopharyngeal carriage of *H influenzae* type b by undertaking annual cross-sectional surveys of a sample of KHDSS residents of all ages, selected at random from the KHDSS population register once every year from 2009 to 2012, as described elsewhere.^13^ Isolates of *H influenzae* type b from nasopharyngeal swabs were identified in the same way as for sterile-site samples.

### Assessment of serological immunity

We assessed serological immunity to *H influenzae* type b in five cross-sectional samples of children aged 12 years or younger within the study area, consisting of four convenience samples from the Junju, Ngerenya, and Chonyi locations in Kilifi County during 1998, 2000, 2004–05, and 2007,^14^, ^15^ and an age-stratified sample (50 children in each of ten age strata: 0 years, 1 year, 2 years, 3 years, 4 years, 5 years, 6 years, 7 years, 8–9 years, and 10–14 years) selected at random using Stata (version 10.1) from each age strata from the population register of the KHDSS in 2009.

Serum samples were stored at −70°C until they were tested using an ELISA for antibodies to PRP. ELISA was done at the WHO Pneumococcal Reference Laboratory, Institute of Child Health, London, UK. Methods were as documented elsewhere,^16^ but with the following alteration: HbOHA antigen (National Institute for Biological Standards and Controls, Hertfordshire, UK) was used at 3 mg/mL. Test, control, and reference (lot 1983; US Food and Drug Administration) serum samples were incubated at 37°C for 1 h. The antibody-binding reaction was monitored by absorbance readings at 410 nm and 630 nm. We determined anti-PRP concentrations by referring to a standard curve generated from the reference wells using four-parameter sigmoid curve fitting. Median values were reported for test serum samples displaying non-parallelism to this curve. Values below the lower limit of quantitation (0·09 mg/mL) were reported as 0·05 mg/mL.

### Statistical analysis

For population-based analyses, we designated Jan 1, 2000, through to Dec 31, 2001, as the prevaccine era and Jan 1, 2004, through to Dec 31, 2014, as the routine-use era, to allow time for sufficient vaccine uptake, given that the Hib vaccine was introduced without a catch-up campaign. We calculated the incidence of invasive *H influenzae* type b disease as the number of KHDSS residents admitted to KCH and confirmed by sterile-site culture to have *H influenzae* type b infection, divided by the resident population at the midpoint of each observation period. We calculated the incidence of *H influenzae* type b meningitis as the number of KHDSS residents admitted to KCH with culture-confirmed *H influenzae* type b (from any sterile site) who met a definition of probable meningitis (CSF white cell count ≥50 × 10^6^ cells/L or a ratio of CSF glucose to plasma glucose of <0·1), divided by the resident population at the midpoint of each observation period. We calculated the incidence rate ratio (IRR) by using Poisson regression for specific age groups and observation periods. We calculated vaccine effectiveness as 1 minus the IRR, expressed as a percentage.

We excluded data from 2004–05 from calculations related to meningitis because a transient change in lumbar puncture clinical practice occurred during this period, and we did not analyse meningitis data after 2010 because pneumococcal conjugate vaccine was introduced in 2011, which was expected to reduce the incidence of probable meningitis. We categorised PRP antibody concentrations according to putative threshold protective concentrations, and calculated geometric mean concentrations by year and age category. Pre-2009 serosurveys did not include children aged 13 years or older, so we excluded data from children who were 13 years or older in 2009 from the serological immunity analyses. We present the decline in PRP antibody concentration and reverse cumulative distribution curves according to age category and year.

We did all statistical analyses using Stata, versions 11.2 and 12.0.

### Role of the funding source

The funders of the study had no role in study design; in the collection, analysis, and interpretation of data; or the writing of the report. The corresponding author had full access to all the data in the study and had final responsibility for the decision to submit for publication.

## Results

40 482 children younger than 13 years resident in KHDSS were admitted to KCH between 2000 and 2014, 38 206 (94%) of whom had their blood cultured. Although the number of cases of invasive *H influenzae* type b disease declined after vaccine introduction, the site of culture, sex of patients, and syndrome-specific mortality did not change substantially between the pre-vaccine and routine-use eras (table 1). In children younger than 5 years, the median age of infection with *H influenzae* type b was 10 months (IQR 5–24) in the prevaccine era and 10·5 months (4–23) in the routine-use era. HIV status was determined for 25 (68%) of the 37 KHDSS children with invasive *H influenzae* type b disease from 2003 to 2014. Four (16%) of the 25 children with *H influenzae* type b disease had HIV infection (three in 2003 and one in 2008). For comparison, HIV prevalence in all paediatric admissions to KCH in 2005–14 was 4·3% (807 of 18 767 admissions in whom HIV results were available).

**Figure 1:**
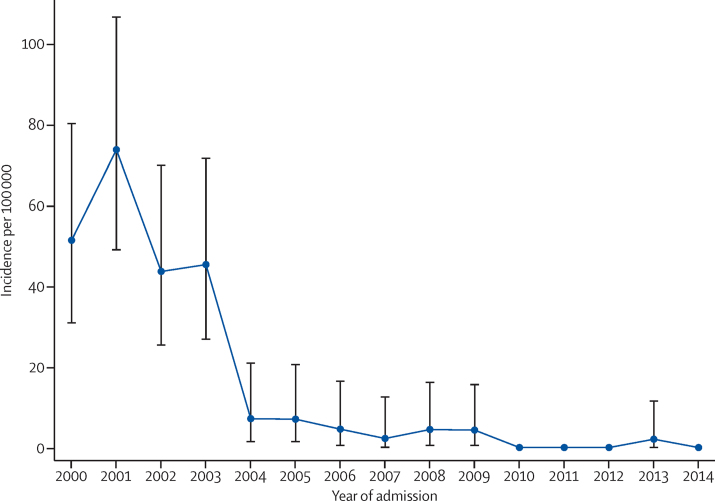
Incidence of invasive *Haemophilus influenzae* type b disease in children younger than 5 years in the Kilifi Health and Demographic Surveillance System, 2000–14 Hib vaccine was introduced in November, 2001. Error bars show 95% CI.

The mean annual incidence of invasive *H influenzae* type b disease in children younger than 5 years in Kilifi was 62·6 per 100 000 (95% CI 46·0–83·3) in the pre-vaccine era (2000–01) and 4·5 per 100 000 (2·5–7·5) in the routine-use era (2004–14; figure 1), which translates to a vaccine effectiveness of 93% (95% CI 87–96; table 2). Incidences of invasive *H influenzae* type b disease and meningitis in the early introduction era (2002–03) were significantly higher than in later periods in children younger than 5 years, but rates of invasive *H influenzae* type b disease were similar in different periods of routine use (table 2). Incidence of invasive *H influenzae* type b disease in children aged 5–12 years was low in the prevaccine era and remained low in the routine-use era (table 2). In the routine-use era, 13 of the 17 cases of invasive *H influenzae* type b disease occurred in children younger than 36 months (the age before which serological immunity starts to decline and a comparable metric to that presented in the first Kilifi analysis^3^). The incidence of non-type-b invasive *H influenzae* disease (ie, serotype replacement disease) did not increase after introduction of the Hib vaccine (IRR in children aged <5 years, 2000–01 *vs* 2004–14, was 0·71 (95% CI 0·19–2·61; table 1).

A previous study^17^ showed that nasopharyngeal carriage of *H influenzae* type b in children younger than 5 years was 1·7% in 2004, 3 years after Hib vaccine introduction. We obtained nasopharyngeal swabs from 2031 KHDSS residents in the four annual cross-sectional nasopharyngeal swab surveys we did in 2009–12. One (0·2%; identified in the 2012 survey) of 623 children younger than 5 years and two (0·1%; both identified in the 2009 survey) of 1408 individuals aged 5 years or older (mean age 34 years [SD 22·5; range 5–92]), carried *H influenzae* type b in the nasopharynx.

**Figure 2:**
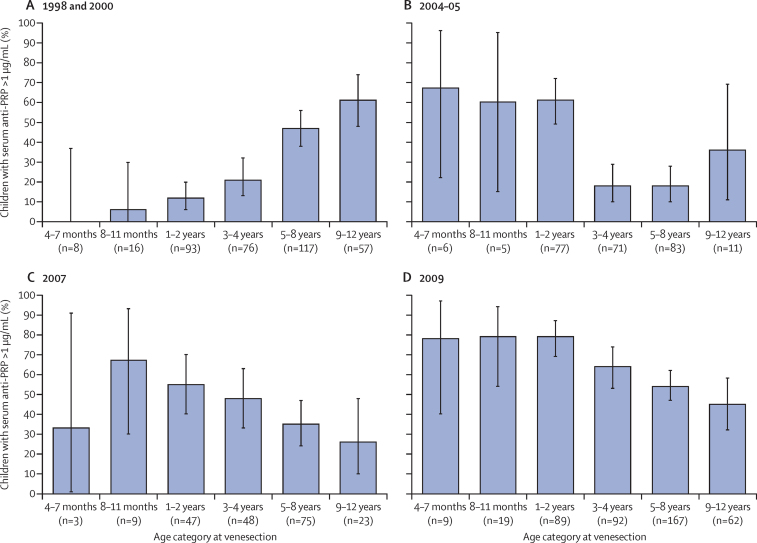
Children with anti-PRP concentrations of >1 μg/mL, by age group and survey year Data for 1998 and 2000 combined (A), 2004–05 (B), 2007 (C), and 2009 (D). The proportion of children aged 4–7 months in (A) is 0%. Error bars show 95% CI. PRP=polyribosylribitol phosphate.

**Figure 3:**
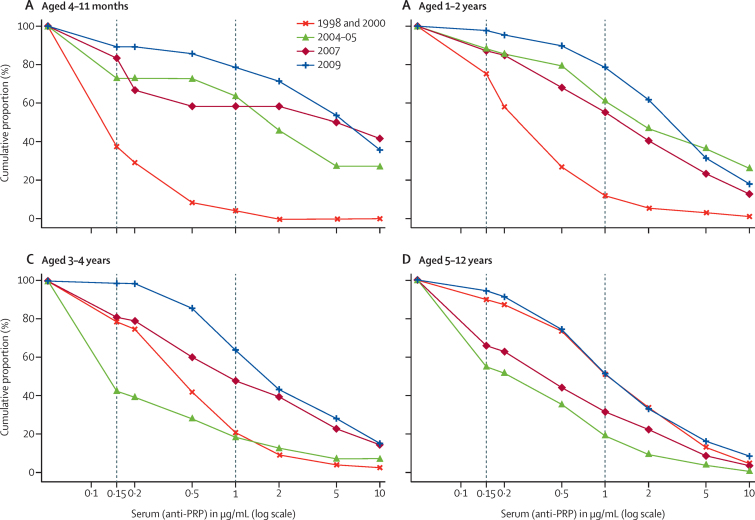
Reverse cumulative proportions of children with anti-PRP concentrations that exceed thresholds, by age group and survey year Data for children aged 4–11 months (A) 1–2 years (B), 3–4 years (C), and 5–12 years (D). Vertical lines indicate thresholds for short-term (0·15 μg/mL) and long-term (1 μg/mL) protection against invasive *H influenzae* type b disease.

To assess serological immunity, we tested available stored serum samples from children younger than 12 years from four convenience samples (367 samples from 1998 and 2000, 253 samples from 2004–05, and 205 samples from 2007), and a further 438 samples from our age-stratified, random sample of KHDSS children in 2009. The pattern of immunity in children in the community is shown in Figure 2, Figure 3 and the appendix. The proportion of children with an anti-PRP concentration of greater than 1 mg/mL (the putative threshold for long-term protection from invasive *H influenzae* type b disease) increased with age in the prevaccine surveys in 1998 and 2000, from very low proportions of infants (one [4%] of 24]) aged 4–7 months and 8–11 months (those at highest risk of invasive *H influenzae* type b disease) up to 35 (61%; 95% CI 48–74) of 57 children aged 9–12 years (figure 2).^18^ After Hib vaccine introduction, a large proportion of children had protective concentrations of antibody (92 [79%; 95% CI 70–86] of 117 children aged 4–35 months had long-term protective anti-PRP concentrations in 2009) and the proportion with long-term protective anti-PRP concentrations did not start to decline until after 36 months of age (figure 2). We noted a similar pattern when assessing the geometric mean concentrations (appendix).

The proportions of children of various ages exceeding anti-PRP concentrations in the years before and after vaccine introduction are shown in the reverse cumulative distribution (RCD) curves (figure 3). The proportions of children aged 4 months to 2 years exceeding anti-PRP concentrations of 0·15 μg/mL and 1 mg/mL were almost uniformly greater in the years after vaccine introduction than in the prevaccine surveys (ie, the RCD curves shift up and to the right after vaccine introduction). In children aged 3–4 years, serological protection declined in 2004–05, but by 2007, population immunity was greater than in prevaccine years. Children aged 5–12 years had a transient decline in serological protection during 2004–05 and 2007, but the RCD curves suggest the same extent of population protection in 2009 as in prevaccine years.

## Discussion

We report sustained control of paediatric invasive *H influenzae* type b disease, to the point of near elimination, in Kilifi, Kenya, in the 13 years after the introduction of Hib vaccine into the routine infant vaccination schedule without a booster dose. Kenya was one of three countries in Africa that were first to include the Hib vaccine in their routine childhood immunisation programme, and this study provides evidence of a robust and durable effect of the vaccine programme. Worldwide, 46 of 54 high-income countries give a booster dose of Hib vaccine, whereas booster doses are used by only six of 78 low-income and lower-middle-income countries and 22 of 57 upper-middle-income countries.^4^ This disparity is a reflection of the fact that support from Gavi, the Vaccine Alliance, for introduction of the Hib vaccine in the poorest countries of the world, which began in 2000, is aligned with the WHO recommendation for routine infant vaccination with Hib as a three-dose primary series.

After introduction of the Hib vaccine, a marked reduction in *H influenzae* type b disease has been documented in many developed and developing regions; however, opportunities to examine the sustainability of the vaccine impact in the absence of a booster dose have been scarce.^1^ Our results are consistent with findings in western regions of The Gambia of near elimination of paediatric *H influenzae* type b meningitis 14 years after introduction of Hib vaccine administered at 2, 3, and 4 months of age and in the absence of a booster dose.^8^ As in The Gambia, our study occurred in a setting with high vaccine coverage: in the KHDSS, coverage with three doses of the Hib vaccine was 91% in children aged 12 months in 2002, 88% in children aged 9–23 months in 2004, 95% in children aged 12 months in 2007, and 93% in 2013 in those aged 12–23 months resident in the KHDSS since birth who had vaccine cards available (Scott JAG, unpublished).^19^, ^20^ Additional evidence of sustained disease control in the absence of a booster dose is provided by data from South America, where the incidence of *H influenzae* type b meningitis 6–10 years after vaccination introduction was similar in four countries, two of which used a booster dose and two of which did not.^21^

By contrast, evidence of waning immunity has prompted three countries—the UK, Mexico, and South Africa—to add a booster dose of Hib vaccine, after they initially recommended only a primary series. In response to low levels of anti-PRP concentrations, persistence of *H influenzae* type b nasopharyngeal carriage, and rising rates of invasive disease, the UK introduced a booster dose of Hib vaccine for children aged 12–15 months in 2006, 10 years after introducing infant Hib vaccine with a catch-up campaign but without a booster dose.^5^ In Mexico, in 2006, results of a cross-sectional study showed that only 40–50% of 110 children aged 12–23 months had anti-PRP concentrations greater than 1 mg/mL.^7^ 92% of these children had received the full Hib vaccine primary course given as the combination pentavalent vaccine, the same combination vaccine as is used in Kenya. On the basis of these results, Mexico, an upper-middle-income country, introduced Hib booster vaccine in 2007. In 2010, 11 years after introducing Hib vaccine as a three-dose primary series, South Africa also introduced a booster dose of the combination vaccine containing Hib. Although the booster dose was primarily for polio prevention, it was hoped that the booster would reduce the number of Hib vaccine failures in South African children (135 [51%] of 263 cases of invasive *H influenzae* type b disease in 2003–09 were classified as being the result of vaccine failures, of which 55% occurred in children aged 18 months or older).^22^ In 2015, a resurgence of invasive *H influenzae* type b disease was reported in eastern regions of The Gambia where coverage with three doses of Hib vaccine was 91% in children aged 12 months, suggesting that a three-dose primary series in the absence of a booster dose might not be providing sustained disease control in this setting.^23^ The reason for this resurgence was unclear, but the authors of the report speculated that it could have been related to waning immunity, continued transmission, or a change in malaria prevalence.

The reason a booster dose is needed to achieve sustained control of disease in some settings but not others remains unclear. Tetanus-toxoid-conjugated Hib vaccine is used widely in developing countries—both in settings with sustained control of *H influenzae* type b disease and in those with evidence of waning immunity—so the vaccine formulation is unlikely to explain the different patterns of disease. Vaccinated individuals are less likely to be carriers of *H influenzae* type b and are therefore less likely to transmit the infection. However, reductions in carriage and transmission also result in fewer opportunities for natural acquisition of anti-*H influenzae* type b antibodies or for boosting of such antibodies. This might have been the reason anti-*H influenzae* type b antibodies declined in adults after routine use of Hib vaccine in children in the UK.^24^ In The Gambia from 1997 to 2002, introduction and widespread use of Hib vaccine was associated with a decline in nasopharyngeal carriage of *H influenzae* type b from 12% to 0·25% in children younger than 5 years.^25^ In 2010, oropharyngeal *H influenzae* type b carriage, as detected by culture, was estimated to be 0·9% in children aged 12–23 months in eastern regions of The Gambia.^8^ This is slightly higher than the carriage prevalence detected by culture of nasopharyngeal swabs reported herein. Culture of oropharyngeal swabs and PCR-based methods might be more sensitive for the detection of *H influenzae* type b than are nasopharyngeal swabs;^26^, ^27^ however, carriage in KHDSS residents was also low when PCR for *H influenzae* type b was done on both oropharyngeal and nasopharyngeal swabs collected from children aged 2–59 months enrolled as controls in a multisite study of pneumonia aetiology in 2011–13 (three [<1%] of 856 children; Hammitt LL and Scott JAG, unpublished). Although older children and adults can serve as a reservoir for transmission, the prevalence of *H influenzae* type b carriage was very low in these age groups in Kilifi. On the basis of these data, the opportunities for natural boosting in Kilifi are rare, as is the risk of exposure.

In Kilifi, in the years after vaccine introduction, naturally acquired antibody has been replaced by vaccine-induced antibody. For infants and young children, this has meant greater serological protection, with 79% (95% CI 59–92) of children aged 4–11 months (historically at greatest risk of invasive disease) now having concentrations greater than 1 mg/mL, the threshold associated with long-term *H influenzae* type b-specific protection.^18^ Older children, who in the prevaccine era had naturally acquired immunity, went through a transitional period (2004–05 and 2007) during which both geometric mean concentrations and proportions exceeding protective thresholds were lower than in the prevaccine era. As surveys of older children started to include those who had been vaccinated, and vaccine-induced antibody was persisting into late childhood, measures for children aged 5–12 years resumed their prevaccine levels by 2009. In essence, the vaccine has led to improvements in population immunity in the youngest, highest-risk age groups without compromising immunity in older children. The low number of serosurvey participants in the youngest age groups is a limitation of these data. Although the 2009 survey participants were selected at random from KHDSS records, earlier surveys were convenience samples, which might have resulted in imbalances in representation of children in KHDSS as a whole. Additionally, the longer duration of freezing could have degraded antibody in older samples. These limitations notwithstanding, these results are promising for the prospect of continued effectiveness of Hib vaccine against invasive disease in older children and for maintenance of herd immunity in this setting. However, continued observation is needed, because the proportion of older children (aged 9–12 years) with anti-PRP concentrations greater than 1 mg/mL in Kilifi in 2009 (28 [45%] of 62 children) was similar to eastern regions of The Gambia (55% of 9–14 year-olds), where a resurgence in disease has been noted.

Our immunogenicity data are similar to other studies' data from low-income or lower-middle-income countries. In vaccine trials in Niger and Nepal, 83–88% and 100% of infants, respectively, had post-primary vaccination concentrations of anti-PRP above 1 mg/mL, declining to 67–75% and 64%, respectively, by late infancy.^28^, ^29^ In Mali, 2 years after vaccine introduction and with coverage at 81%, 82% of infants aged 6–7 months had anti-PRP concentrations greater than 1 mg/mL.^30^ In the same setting the following year, antibody decline did not begin until after 2 years of age.^31^ Our results also lend support to the previously observed findings that children in developing countries generate higher anti-PRP concentrations in response to vaccination than those in developed countries such as the UK.^32^ Proposed reasons for this include higher background environmental *H influenzae* type b exposure in developing countries and exposure to bacterial polysaccharides that cross-react with the PRP capsular polysaccharide of *H influenzae* type b.^33^, ^34^, ^35^, ^36^ Exposure to potentially cross-reactive organisms such as *Escherichia coli* or serogroup 6 pneumococci is likely to be higher in developing countries without access to improved water and sanitation or with higher overall pneumococcal carriage prevalence and density than in high-income or middle-income settings.^13^, ^37^, ^38^, ^39^ The level and effects of this exposure could change with improvements in water and sanitation and expanding use of pneumococcal conjugate vaccines. Continued surveillance will monitor whether effective control of disease persists or whether shifts in epidemiology (eg, disease occurring in older children, fewer opportunities for natural boosting) will necessitate a booster dose.

The findings reported herein do not address the possible benefit of a booster dose of Hib vaccine in settings with different epidemiological characteristics from Kilifi (eg, higher HIV prevalence, lower vaccine coverage, exposure to highly unvaccinated populations that might have high rates of carriage). Because the reasons why some settings require a booster dose of Hib vaccine to maintain control of disease and others do not are not well understood, local epidemiological data is vital to guide vaccine policy. In the absence of long-standing surveillance for invasive *H influenzae* type b disease, low *H influenzae* type b carriage prevalence in large studies that include both children and adults and use sensitive methods of detection, or high prevalence of protective antibody concentrations throughout the ages of highest risk for *H influenzae* type b disease, would provide evidence of ongoing protection.

Over the past 25 years, 189 countries, including 73 countries eligible for support from Gavi, the Vaccine Alliance, have introduced a Hib-containing vaccine into their national immunisation programme for children. A booster dose of Hib vaccine is recommended in most high-income countries, whereas most low-income countries have followed the WHO EPI schedule, which does not include a booster dose. In this study, we found that use of Hib vaccine according to the EPI schedule led to near elimination of paediatric invasive *H influenzae* type b disease, with no evidence of resurgent disease in older children in whom immunity might be expected to wane without a booster dose. Indeed, immunogenicity data show that immunity persists through the age of greatest risk of disease for most children and that antibody concentrations in older children, although lower than in young children, are similar to concentrations reported in the prevaccine era. In sum, we found no evidence to support introduction of a booster dose of Hib vaccine into the Kenyan EPI at this time.

## Figures and Tables

**Table 1:** Invasive *Haemophilus influenzae* disease in children aged 0–12 years in the Kilifi Health and Demographic Surveillance System admitted to the Kilifi County Hospital, 2000–14

			**2000**	**2001**	**2002**	**2003**	**2004**	**2005**	**2006**	**2007**	**2008**	**2009**	**2010**	**2011**	**2012**	**2013**	**2014**
**Age <5 years**																	
Admissions			3660	3591	3282	2776	2716	2317	2455	2130	1986	2131	1905	1642	1494	1001	1473
With blood culture			3613 (99%)	3533 (98%)	3200 (98%)	2710 (98%)	2631 (97%)	2219 (96%)	2366 (96%)	1991 (93%)	1841 (93%)	1996 (94%)	1747 (92%)	1528 (93%)	1331 (89%)	904 (90%)	1335 (91%)
With lumbar puncture			939 (26%)	866 (24%)	936 (29%)	578 (21%)	351 (13%)	482 (21%)	693 (28%)	611 (29%)	553 (28%)	548 (26%)	532 (28%)	433 (26%)	410 (27%)	260 (26%)	390 (26%)
With probable bacterial meningitis			33 (1%)	40 (1%)	52 (2%)	24 (1%)	20 (1%)	24 (1%)	25 (1%)	26 (1%)	18 (1%)	16 (1%)	18 (1%)	18 (1%)	11 (1%)	11 (1%)	5 (<1%)
Culture-confirmed *H influenzae* disease			24 (<1%)	30 (<1%)	21 (<1%)	22 (<1%)	4 (<1%)	5 (<1%)	2 (<1%)	2 (<1%)	5 (<1%)	2 (<1%)	1 (<1%)	0	3 (<1%)	2 (<1%)	3 (<1%)
	Types a, c, d, e, f		2 (8%)	1 (3%)	1 (5%)	0	1 (25%)	1 (20%)	0	1 (50%)	2 (40%)	0	0	0	2 (67%)	1 (50%)	1 (33%)
	Non-capsular		3 (13%)	1 (3%)	3 (14%)	4 (18%)	0	1 (20%)	0	0	1 (20%)	0	1 (100%)	0	1 (33%)	0	2 (67%)
	Type b		19 (79%)	28 (93%)	17 (81%)	18 (82%)	3 (75%)	3 (60%)	2 (100%)	1 (50%)	2 (40%)	2 (100%)	0	0	0	1 (50%)	0
		*H influenzae* type b cultured in CSF	4 (21%)	13 (46%)	8 (47%)	8 (44%)	1 (33%)	1 (33%)	0	0	1 (50%)	1 (50%)	0	0	0	0	0
		Age <24 months	16 (84%)	19 (68%)	10 (59%)	10 (56%)	2 (67%)	1 (33%)	0	1 (100%)	1 (50%)	2 (100%)	0	0	0	1 (100%)	0
		Boys	7 (37%)	15 (54%)	6 (35%)	7 (39%)	2 (67%)	1 (33%)	1 (50%)	1 (100%)	0	1 (50%)	0	0	0	1 (100%)	0
		Died during the episode	3 (16%)	8 (29%)	5 (29%)	6 (33%)	0	1 (33%)	0	1 (100%)	1 (50%)	0	0	0	0	1 (100%)	0
**Age 5–12 years**																	
Admissions			459	398	377	416	373	425	419	369	356	395	436	413	324	283	480
With blood culture			448 (98%)	378 (95%)	361 (96%)	394 (95%)	328 (88%)	371 (87%)	376 (90%)	326 (88%)	308 (87%)	331 (84%)	380 (87%)	347 (84%)	274 (85%)	226 (80%)	413 (86%)
With lumbar puncture			74 (16%)	55 (14%)	68 (18%)	54 (13%)	41 (11%)	37 (9%)	56 (13%)	44 (12%)	50 (14%)	46 (12%)	88 (20%)	65 (16%)	71 (22%)	81 (29%)	96 (20%)
With probable bacterial meningitis			7 (2%)	5 (1%)	6 (2%)	10 (2%)	5 (1%)	1 (<1%)	1 (<1%)	5 (1%)	4 (1%)	3 (1%)	4 (1%)	3 (1%)	1 (<1%)	4 (1%)	1 (<1%)
Culture-confirmed *H influenzae* disease			1 (<1%)	2 (<1%)	2 (<1%)	2 (<1%)	1 (<1%)	1 (<1%)	1 (<1%)	0	0	1 (<1%)	1 (<1%)	1 (<1%)	0	0	1 (<1%)
	Types a, c, d, e, f		0	0	1 (50%)	0	0	0	1 (100%)	0	0	0	0	0	0	0	0
	Non-capsular		1 (100%)	0	0	0	0	1 (100%)	0	0	0	0	0	1 (100%)	0	0	1 (100%)
	Type b		0	2 (100%)	1 (50%)	2 (100%)	1 (100%)	0	0	0	0	1 (100%)	1 (100%)	0	0	0	0
		*H influenzae* type b cultured in CSF	0	1 (50%)	1 (100%)	1 (50%)	1 (100%)	0	0	0	0	1 (100%)	1 (100%)	0	0	0	0
		Boys	0	1 (50%)	1 (100%)	1 (50%)	0	0	0	0	0	1 (100%)	1 (100%)	0	0	0	0
		Died during the episode	0	1 (50%)	1 (100%)	1 (50%)	0	0	0	0	0	0	0	0	0	0	0

Data are N and n (%). CSF=cerebrospinal fluid.

**Table 2:** Incidence (per 100 000) of invasive *Haemophilus influenzae* type b disease and meningitis, and probable bacterial meningitis, in children aged 0–12 years in the Kilifi Health and Demographic Surveillance System, 2000–14

		**Age <2 years**		**Age <5 years**		**Age 5–12 years**	
		N	Incidence (95% CI)	N	Incidence (95% CI)	N	Incidence (95% CI)
**Invasive *H influenzae* type b disease**							
2000–01		35	117·1 (81·6–162·9)	47	62·6 (46·0–83·3)	2	4·0 (0·5–14·6)
2002–03		25	79·4 (51·4–117·2)	34	44·6 (31·0–61·8)	3	2·9 (0·6–8·6)
2004–14		11	8·6 (4·3–15·3)	14	4·5 (2·5–7·5)	3	1·7 (0·3–4·8)
	2004–05	5	14·5 (4·7–33·8)	6	7·1 (2·6–15·4)	1	1·9 (0·1–10·3)
	2006–09	5	6·7 (2·2–15·7)	7	3·9 (1·6–8·0)	1	1·6 (0·0–8·8)
	2010–14	1	5·1 (0·1–28·3)	1	2·1 (0·1–11·5)	1	1·6 (0·0–8·6)
Incidence rate ratio							
	2000–01 *vs* 2002–03	..	0·67 (0·41–1·13)	..	0·71 (0·46–1·10)	..	0·73 (0·12–4·34)
	2000–01 *vs* 2004–14	..	0·07 (0·04–0·14)	..	0·07 (0·04–0·13)	..	0·41 (0·07–2·44)
***H influenzae* type b meningitis**							
2000–01		14	46·8 (25·6–78·6)	17	22·7 (13·2–36·3)	1	2·0 (0·5–11·2)
2002–03		12	38·1 (19·7–66·6)	15	19·1 (10·7–31·4)	1	2·0 (0·1–11·0)
2006–10		2	5·3 (0·7–19·2)	2	2·2 (0·3–7·9)	2	1·6 (0·2–5·6)
Incidence rate ratio							
	2000–01 *vs* 2002–03	..	0·81 (0·38–1·76)	..	0·84 (0·42–1·68)	..	0·97 (0·06–15·57)
	2000–01 *vs* 2006–10	..	0·11 (0·03–0·50)	..	0·09 (0·02–0·42)	..	0·77 (0·07–8·54)
**Probable bacterial meningitis**							
2000–01		61	204·1 (156·1–262·2)	73	97·3 (76·3–122·3)	15	15·3 (8·6–25·3)
2002–03		67	212·9 (165·0–270·3)	76	96·5 (76·0–120·8)	17	16·6 (9·7–26·6)
2006–10		80	85·4 (67·7–106·3)	103	45·3 (37·0–55·0)	17	5·5 (3·2–8·8)
Incidence rate ratio							
	2000–01 *vs* 2002–03	..	1·04 (0·74–1·48)	..	0·99 (0·72–1·37)	..	1·08 (0·54–2·17)
	2000–01 *vs* 2006–10	..	0·41 (0·30–0·58)	..	0·47 (0·35–0·63)	..	0·36 (0·18–0·72)

N is the number of children with the disease during the corresponding timeframe.
