# Supplementary material for: Effect of Haemophilus influenzae type b vaccination without a booster dose on invasive H influenzae type b disease, nasopharyngeal carriage, and population immunity in Kilifi, Kenya: a 15-year regional surveillance study
Source: Lancet Glob Health. 2016 Feb 5;4(3):e185–94. doi: 10.1016/S2214-109X(15)00316-2 (PMC4763163; doi:10.1016/S2214-109X(15)00316-2)
Supplement: Supplementary appendix [file mmc1.pdf]

THE LANCET Global Health

Supplementary appendix

This appendix formed part of the original submission and has been peer reviewed. We post it as supplied by the authors.

Supplement to: Hammitt LL, Crane RJ, Karani A, et al. Effect of *Haemophilus influenzae* type b vaccination without a booster dose on invasive *H influenzae* type b disease, nasopharyngeal carriage, and population immunity in Kilifi, Kenya: a 15-year regional surveillance study. *Lancet Glob Health* 2016; published online Feb 4. http://dx.doi.org/10.1016/S2214-109X(15)00316-2.

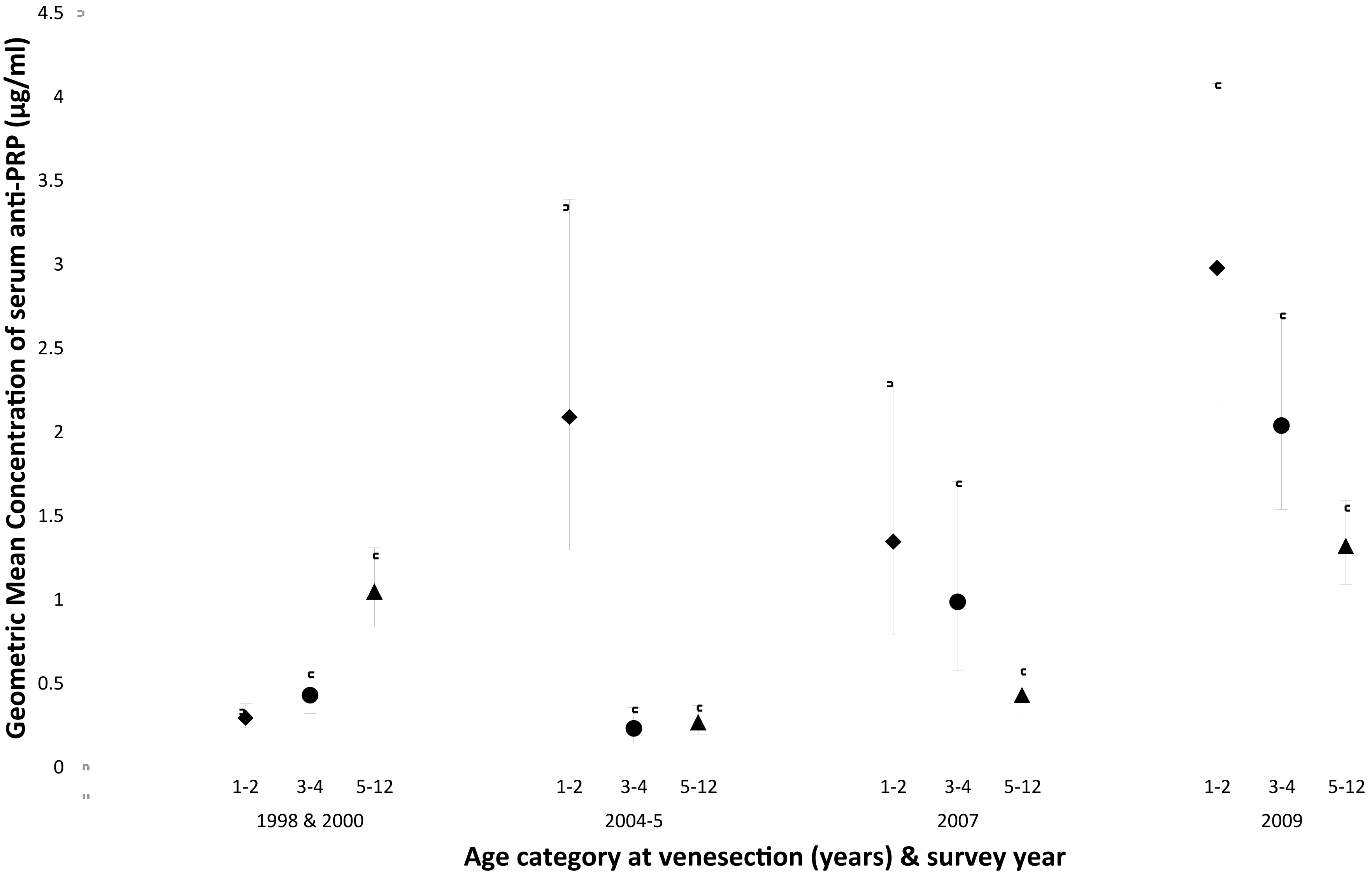

Geometric Mean Concentration of serum anti-PRP (µg/ml)
4.5
4
3.5
3
2.5
2
1.5
1
0.5
0
1-2
3-4
5-12
1998 & 2000
1-2
3-4
5-12
2004-5
1-2
3-4
5-12
2007
1-2
3-4
5-12
2009
Age category at venesection (years) & survey year
